# The first record of *Caenis rivulorum* (Ephemeroptera: Caenidae) from Japan

**DOI:** 10.3897/BDJ.9.e67413

**Published:** 2021-07-08

**Authors:** Tatsushi Takayanagi, Kazunori Yoshizawa

**Affiliations:** 1 Systematic Entomology, Graduate School of Agriculture, Hokkaido University, Sapporo, Japan Systematic Entomology, Graduate School of Agriculture, Hokkaido University Sapporo Japan

**Keywords:** Ephemeroptera, mayflies, Caenidae, *
Caenis
*, Japan, Hokkaido, COI

## Abstract

**Background:**

*Caenis rivulorum* Eaton, 1884 is widely distributed and has been reported from a wide range in the Palearctic Region.

**New information:**

We report this species from Japan for the first time, from five localities of Hokkaido, based on morphology and molecular data.

## Introduction

The genus *Caenis* Stephens, 1836 (Ephemeroptera: Caenidae) is a cosmopolitan group of mayflies with more than 140 described species and is one of the most diversified genus of mayflies (Barber-James et al. 2007, Barber-James et al. 2013). *Caenis rivulorum* Eaton, 1884 is widely distributed in the Palearctic Region and has been reported from a wide range in Europe (Malzacher 1984, Malzacher 1986, Bauernfeind and Soldan 2012), Russia including Far East and Sakhalin (Tiunova 2007, Tiunova 2009, Tiunova 2012), the north-east part of China (Zhou and Zheng 2004) and western part of Mongolia (Erdenee et al. 2016). However, it has not been recorded from Japan to date. In this paper, we report this species from Hokkaido, the northern part of Japan, for the first time. In addition, brief comments are given for the study of the genus in Japan.

## Materials and methods

### Morphological observations

**Figure 1.**
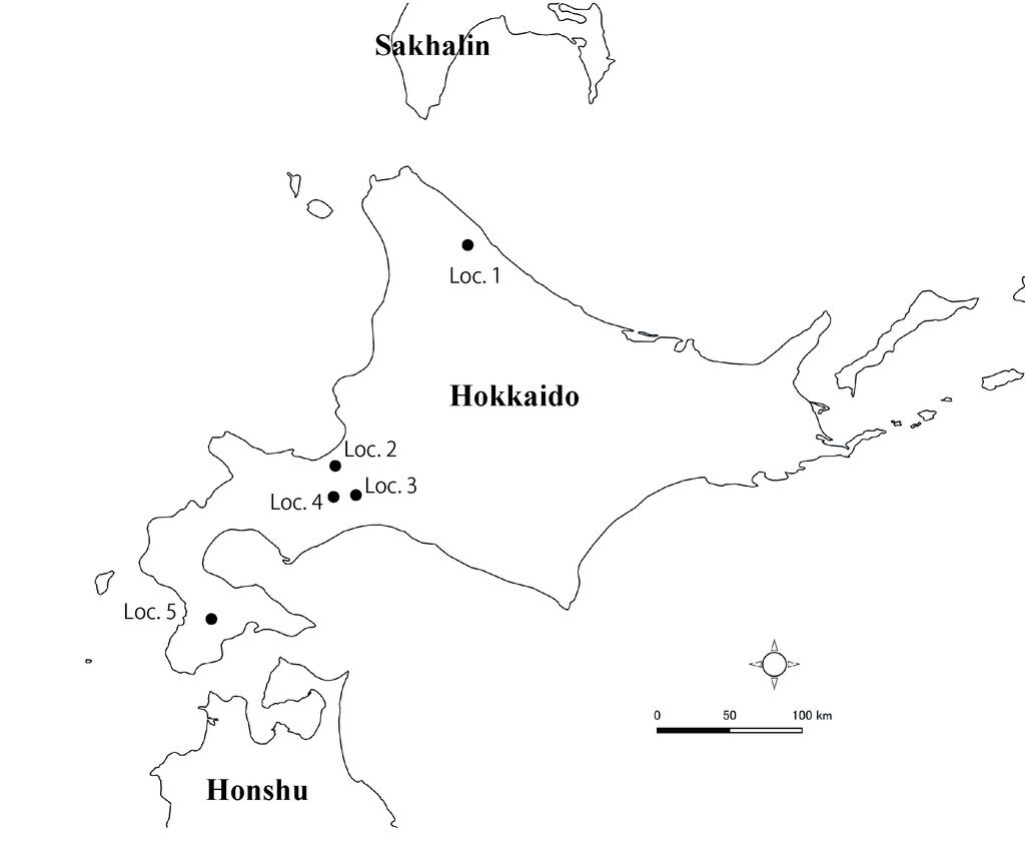
Locations of collecting localities. **Loc. 1** Usotan, Hamatonbetsu-cho, Hokkaido, Japan. N45.046779, E142.361674; **Loc. 2** Kita 17 jo, Nishi 9 chome, Kita-ku, Sapporo-shi, Hokkaido, Japan. N43.079981, E141.338084; **Loc. 3** Rankoshi, Chitose-shi, Hokkaido, Japan. N42.809656, E141.574878; **Loc. 4** Poropinai, Chitose-shi, Hokkaido, Japan. N42.801191, E141.326650; **Loc. 5** Shirooka, Assabu-cho, Hokkaido, Japan. N41.832767, E140.332714.

Adult mayflies were collected from five distinct localities of Hokkaido in 2019 and 2020 (Fig. 1). All of them were collected by using a light trap or collected around street lights. They were stored in 80% ethanol. Genital structures were observed under a Zeiss Axiophoto microscope (CarlZeissAG, Jena, Germany). Digital images of specimens were captured using AxioCam ERc 5s (CarlZeissAG, Jena, Germany) attached to the microscope. The genital structures were observed after processing in Proteinase K and stained with acid fuchsin. The mesonotum structure was observed after processing in potassium hydroxide (KOH). Eggs were extracted from a female imago from Loc. 2 and dehydrated through a series of increasing concentrations of ethanol (80%, 90%, 95%) before immersion in absolute ethanol, 10 minutes each, then placed in hexamethyldisilizane (HMDS) for 15 minutes. After 30 minutes air drying, eggs were mounted on stubs with conductive sticky tape and sputter-coated with gold-palladium in an ion-sputtering device MSP-20-MT Automatic Magnetron Sputter (Vacuum Device, Ibaraki, Japan). Finally, the eggs were observed through scanning electron microscopes JSM-5310LV (JEOL, Tokyo, Japan). The terms of morphology followed Bauernfeind and Soldan (2012) and the terms of genital morphology followed Malzacher (1991).

### Molecular methods

Total DNA was extracted from the specimens using Qiagen DNeasy Tissue Kit (QIAGEN, Venlo, The Netherlands). DNA of male adults was extracted from the whole abdomen. The exoskeleton was kept for further observation. DNA of female adults was extracted from the thoracic muscle tissues. The barcode region of COI gene was amplified by the PCR method using the primer set, LCO1490 and HCO2198 (Folmer et al. 1994). The PCR cycle was: 1 min at 94°C followed by 40 cycles of 30 sec at 94°C, 30 sec at 45°C, 1 min at 68°C, then 1 min at 68°C using BIO RAD T-100 Thermal Cycler (Bio-Rad Laboratories, California, USA). PCR products were purified using Fast Gene Gel/PCR Extraction kit (Nippon Genetics, Tokyo, Japan). The purified DNA was sequenced using Beckman Coulter CEQ2000XL (Beckman Coulter, California, USA). The COI sequences of three male adults and five female adults from Loc. 1, 2 and 5 were deposited to GenBank under accession numbers as noted in Materials, Taxon treatment.

### Sequence analysis

All sequence data were aligned automatically using MAFFT v.7.429 (Katoh et al. 2002) then visually cross-checked using Mesquite software (Maddison and Maddison 2019). Pairwise distances were calculated using MEGAX (Kumar et al. 2018, Stecher et al. 2020). A substitution model was selected using ModelFinder (Kalyaanamoorthy et al. 2017). Maximum Likelihood analysis was conducted using IQ-Tree (Nguyen et al. 2014). Ultra-fast Bootstrap was conducted (Hoang et al. 2018) with 1000 replications. The Maximum Likelihood tree was edited in the FigTree v. 1.4.4 (http://tree.bio.ed.ac.uk/software/figtree). The sequences of the related taxa were obtained from an article proposing DNA barcode library, based on taxonomically well-curated specimens (Morinière et al. 2017).

## Taxon treatments

### Caenis rivulorum

Eaton, 1884

D9675F21-F901-5CC5-BF59-98D4EC9B0468

Caenis dimidiata var. rivulorum Eaton, 1884 in Eaton (1884), Trans. Linn. Soc. London, 2nd Ser. 3 (2): 143 (Type locality: Thorncombe, Dorset, England).
Caenis rivulorum Eaton, 1884 in Eaton (1888), Trans. Linn. Soc. London, 2nd Ser. 3 (2): 320. Change of status.
Caenis nivea Bengtsson, 1917 in Bengtsson 1917, Ent. Tidskr. 38: 2, p. 181–182, synonymised by Saaristo (1966).
Caenis nigroforceps Zhou, Gui & Su, 1997 in Zhou et al. 1997, Entomotaxonomia 19: 270–271, synonymised by Zhou and Zheng (2004).

#### Materials

- **Type status:** Other material. **Occurrence:** recordedBy: Yuto Okita; individualID: im0023_L024; individualCount: 1; sex: male; lifeStage: adult; preparations: abdomen in glycerine, other parts in 99% EtOH; **Taxon:** scientificName: *Caenis rivulorum*; kingdom: Animalia; phylum: Euarthropoda; class: Insecta; order: Ephemeroptera; family: Caenidae; genus: Caenis; specificEpithet: *rivulorum*; scientificNameAuthorship: Eaton; **Location:** country: Japan; stateProvince: Hokkaido; county: Soya-gun; municipality: Hamatonbetsu-cho; locality: Usotan; decimalLatitude: 45.0468; decimalLongitude: 142.3617; **Identification:** identifiedBy: Tatsushi Takayanagi; dateIdentified: 2020; **Event:** samplingProtocol: light trap; eventDate: 2019-07-06; fieldNotes: DNA extraction: LC619650; **Record Level:** collectionCode: Insects
- **Type status:** Other material. **Occurrence:** recordedBy: Yuto Okita; individualID: im0024_L025; individualCount: 1; sex: female; lifeStage: adult; preparations: 99%EtOH; **Taxon:** scientificName: *Caenis rivulorum*; kingdom: Animalia; phylum: Euarthropoda; class: Insecta; order: Ephemeroptera; family: Caenidae; genus: Caenis; specificEpithet: *rivulorum*; scientificNameAuthorship: Eaton; **Location:** country: Japan; stateProvince: Hokkaido; county: Soya-gun; municipality: Hamatonbetsu-cho; locality: Usotan; decimalLatitude: 45.0468; decimalLongitude: 142.3617; **Identification:** identifiedBy: Tatsushi Takayanagi; dateIdentified: 2020; **Event:** samplingProtocol: light trap; eventDate: 2019-07-06; fieldNotes: DNA extraction: LC619651; **Record Level:** collectionCode: Insects
- **Type status:** Other material. **Occurrence:** recordedBy: Tatsushi Takayanagi; individualID: im0025_L026; individualCount: 1; sex: male; lifeStage: adult; preparations: abdomen in glycerine, other parts in 99% EtOH; **Taxon:** scientificName: *Caenis rivulorum*; kingdom: Animalia; phylum: Euarthropoda; class: Insecta; order: Ephemeroptera; family: Caenidae; genus: Caenis; specificEpithet: *rivulorum*; scientificNameAuthorship: Eaton; **Location:** country: Japan; stateProvince: Hokkaido; county: Hiyama-gun; municipality: Assabu-cho; locality: Shirooka; decimalLatitude: 41.8328; decimalLongitude: 140.3327; **Identification:** identifiedBy: Tatsushi Takayanagi; dateIdentified: 2020; **Event:** samplingProtocol: light trap; eventDate: 2019-07-08; fieldNotes: DNA extraction: LC619652; **Record Level:** collectionCode: Insects
- **Type status:** Other material. **Occurrence:** recordedBy: Tatsushi Takayanagi; individualID: im0026_L027; individualCount: 1; sex: female; lifeStage: adult; preparations: 99% EtOH; **Taxon:** scientificName: *Caenis rivulorum*; kingdom: Animalia; phylum: Euarthropoda; class: Insecta; order: Ephemeroptera; family: Caenidae; genus: Caenis; specificEpithet: *rivulorum*; scientificNameAuthorship: Eaton; **Location:** country: Japan; stateProvince: Hokkaido; county: Hiyama-gun; municipality: Assabu-cho; locality: Shirooka; decimalLatitude: 41.8328; decimalLongitude: 140.3327; **Identification:** identifiedBy: Tatsushi Takayanagi; dateIdentified: 2020; **Event:** samplingProtocol: light trap; eventDate: 2019-07-08; fieldNotes: DNA extraction: LC619653; **Record Level:** collectionCode: Insects
- **Type status:** Other material. **Occurrence:** recordedBy: Yuto Okita; individualID: im0027_L028; individualCount: 1; sex: male; lifeStage: adult; preparations: abdomen in glycerine, other parts in 99% EtOH; **Taxon:** scientificName: *Caenis rivulorum*; kingdom: Animalia; phylum: Euarthropoda; class: Insecta; order: Ephemeroptera; family: Caenidae; genus: Caenis; specificEpithet: *rivulorum*; scientificNameAuthorship: Eaton; **Location:** country: Japan; stateProvince: Hokkaido; municipality: Sapporo-shi; locality: Kita-ku, Kita-17, Nishi-9; decimalLatitude: 43.08; decimalLongitude: 141.3381; **Identification:** identifiedBy: Tatsushi Takayanagi; dateIdentified: 2020; **Event:** samplingProtocol: light trap; eventDate: 2019-07-10; fieldNotes: DNA extraction: LC619654; **Record Level:** collectionCode: Insects
- **Type status:** Other material. **Occurrence:** recordedBy: Yuto Okita; individualID: im0028_L029; individualCount: 1; sex: female; lifeStage: adult; preparations: 99% EtOH; **Taxon:** scientificName: *Caenis rivulorum*; kingdom: Animalia; phylum: Euarthropoda; class: Insecta; order: Ephemeroptera; family: Caenidae; genus: Caenis; specificEpithet: *rivulorum*; scientificNameAuthorship: Eaton; **Location:** country: Japan; stateProvince: Hokkaido; municipality: Sapporo-shi; locality: Kita-ku, Kita-17, Nishi-9; decimalLatitude: 43.08; decimalLongitude: 141.3381; **Identification:** identifiedBy: Tatsushi Takayanagi; dateIdentified: 2020; **Event:** samplingProtocol: light trap; eventDate: 2019-07-10; fieldNotes: DNA extraction: LC619655; **Record Level:** collectionCode: Insects
- **Type status:** Other material. **Occurrence:** recordedBy: Yuto Okita; individualID: im0029_L030; individualCount: 1; sex: female; lifeStage: adult; preparations: 99% EtOH; **Taxon:** scientificName: *Caenis rivulorum*; kingdom: Animalia; phylum: Euarthropoda; class: Insecta; order: Ephemeroptera; family: Caenidae; genus: Caenis; specificEpithet: *rivulorum*; scientificNameAuthorship: Eaton; **Location:** country: Japan; stateProvince: Hokkaido; municipality: Sapporo-shi; locality: Kita-ku, Kita-17, Nishi-9; decimalLatitude: 43.08; decimalLongitude: 141.3381; **Identification:** identifiedBy: Tatsushi Takayanagi; dateIdentified: 2020; **Event:** samplingProtocol: light trap; eventDate: 2019-07-10; fieldNotes: DNA extraction: LC619656; **Record Level:** collectionCode: Insects
- **Type status:** Other material. **Occurrence:** recordedBy: Tatsushi Takayanagi; individualID: im0030_L031; individualCount: 1; sex: female; lifeStage: adult; preparations: 99% EtOH; **Taxon:** scientificName: *Caenis rivulorum*; kingdom: Animalia; phylum: Euarthropoda; class: Insecta; order: Ephemeroptera; family: Caenidae; genus: Caenis; specificEpithet: *rivulorum*; scientificNameAuthorship: Eaton; **Location:** country: Japan; stateProvince: Hokkaido; county: Hiyama-gun; municipality: Assabu-cho; locality: Shirooka; decimalLatitude: 41.8328; decimalLongitude: 140.3327; **Identification:** identifiedBy: Tatsushi Takayanagi; dateIdentified: 2020; **Event:** samplingProtocol: light trap; eventDate: 2019-07-08; fieldNotes: DNA extraction: LC619657; **Record Level:** collectionCode: Insects
- **Type status:** Other material. **Occurrence:** recordedBy: Yuto Okita; individualCount: 5; sex: male; lifeStage: adult; preparations: 99% EtOH; **Taxon:** scientificName: *Caenis rivulorum*; kingdom: Animalia; phylum: Euarthropoda; class: Insecta; order: Ephemeroptera; family: Caenidae; genus: Caenis; specificEpithet: *rivulorum*; scientificNameAuthorship: Eaton; **Location:** country: Japan; stateProvince: Hokkaido; county: Soya-gun; municipality: Hamatonbetsu-cho; locality: Usotan; decimalLatitude: 45.0468; decimalLongitude: 142.3617; **Identification:** identifiedBy: Tatsushi Takayanagi; dateIdentified: 2020; **Event:** samplingProtocol: light trap; eventDate: 2019-07-06; **Record Level:** collectionCode: Insects
- **Type status:** Other material. **Occurrence:** recordedBy: Yuto Okita; individualCount: 2; sex: female; lifeStage: adult; preparations: 99% EtOH; **Taxon:** scientificName: *Caenis rivulorum*; kingdom: Animalia; phylum: Euarthropoda; class: Insecta; order: Ephemeroptera; family: Caenidae; genus: Caenis; specificEpithet: *rivulorum*; scientificNameAuthorship: Eaton; **Location:** country: Japan; stateProvince: Hokkaido; county: Soya-gun; municipality: Hamatonbetsu-cho; locality: Usotan; decimalLatitude: 45.0468; decimalLongitude: 142.3617; **Identification:** identifiedBy: Tatsushi Takayanagi; dateIdentified: 2020; **Event:** samplingProtocol: light trap; eventDate: 2019-07-06; **Record Level:** collectionCode: Insects
- **Type status:** Other material. **Occurrence:** recordedBy: Tatsushi Takayanagi; individualCount: 2; sex: male; lifeStage: adult; preparations: 99% EtOH; **Taxon:** scientificName: *Caenis rivulorum*; kingdom: Animalia; phylum: Euarthropoda; class: Insecta; order: Ephemeroptera; family: Caenidae; genus: Caenis; specificEpithet: *rivulorum*; scientificNameAuthorship: Eaton; **Location:** country: Japan; stateProvince: Hokkaido; county: Hiyama-gun; municipality: Assabu-cho; locality: Shirooka; decimalLatitude: 41.8328; decimalLongitude: 140.3327; **Identification:** identifiedBy: Tatsushi Takayanagi; dateIdentified: 2020; **Event:** samplingProtocol: light trap; eventDate: 2019-07-08; **Record Level:** collectionCode: Insects
- **Type status:** Other material. **Occurrence:** recordedBy: Tatsushi Takayanagi; individualCount: many; sex: female; lifeStage: adult; preparations: 99% EtOH; **Taxon:** scientificName: *Caenis rivulorum*; kingdom: Animalia; phylum: Euarthropoda; class: Insecta; order: Ephemeroptera; family: Caenidae; genus: Caenis; specificEpithet: *rivulorum*; scientificNameAuthorship: Eaton; **Location:** country: Japan; stateProvince: Hokkaido; county: Hiyama-gun; municipality: Assabu-cho; locality: Shirooka; decimalLatitude: 41.8328; decimalLongitude: 140.3327; **Identification:** identifiedBy: Tatsushi Takayanagi; dateIdentified: 2020; **Event:** samplingProtocol: light trap; eventDate: 2019-07-08; **Record Level:** collectionCode: Insects
- **Type status:** Other material. **Occurrence:** recordedBy: Yuto Okita; individualCount: 1; sex: male; lifeStage: adult; preparations: 99% EtOH; **Taxon:** scientificName: *Caenis rivulorum*; kingdom: Animalia; phylum: Euarthropoda; class: Insecta; order: Ephemeroptera; family: Caenidae; genus: Caenis; specificEpithet: *rivulorum*; scientificNameAuthorship: Eaton; **Location:** country: Japan; stateProvince: Hokkaido; municipality: Sapporo-shi; locality: Kita-ku, Kita-17, Nishi-9; decimalLatitude: 43.08; decimalLongitude: 141.3381; **Identification:** identifiedBy: Tatsushi Takayanagi; dateIdentified: 2020; **Event:** samplingProtocol: light trap; eventDate: 2019-07-10; **Record Level:** collectionCode: Insects
- **Type status:** Other material. **Occurrence:** recordedBy: Yuto Okita; individualCount: many; sex: female; lifeStage: adult; preparations: 99% EtOH; **Taxon:** scientificName: *Caenis rivulorum*; kingdom: Animalia; phylum: Euarthropoda; class: Insecta; order: Ephemeroptera; family: Caenidae; genus: Caenis; specificEpithet: *rivulorum*; scientificNameAuthorship: Eaton; **Location:** country: Japan; stateProvince: Hokkaido; municipality: Sapporo-shi; locality: Kita-ku, Kita-17, Nishi-9; decimalLatitude: 43.08; decimalLongitude: 141.3381; **Identification:** identifiedBy: Tatsushi Takayanagi; dateIdentified: 2020; **Event:** samplingProtocol: light trap; eventDate: 2019-07-10; **Record Level:** collectionCode: Insects
- **Type status:** Other material. **Occurrence:** recordedBy: Tatsushi Takayanagi; individualCount: many; sex: male; lifeStage: adult; preparations: 99% EtOH; **Taxon:** scientificName: *Caenis rivulorum*; kingdom: Animalia; phylum: Euarthropoda; class: Insecta; order: Ephemeroptera; family: Caenidae; genus: Caenis; specificEpithet: *rivulorum*; scientificNameAuthorship: Eaton; **Location:** country: Japan; stateProvince: Hokkaido; municipality: Chitose-shi; locality: Poropinai; decimalLatitude: 42.801191; decimalLongitude: 141.32665; **Identification:** identifiedBy: Tatsushi Takayanagi; dateIdentified: 2020; **Event:** samplingProtocol: street light; eventDate: 2020-07-15; **Record Level:** collectionCode: Insects
- **Type status:** Other material. **Occurrence:** recordedBy: Tatsushi Takayanagi; individualCount: many; sex: female; lifeStage: adult; preparations: 99% EtOH; **Taxon:** scientificName: *Caenis rivulorum*; kingdom: Animalia; phylum: Euarthropoda; class: Insecta; order: Ephemeroptera; family: Caenidae; genus: Caenis; specificEpithet: *rivulorum*; scientificNameAuthorship: Eaton; **Location:** country: Japan; stateProvince: Hokkaido; municipality: Chitose-shi; locality: Poropinai; decimalLatitude: 42.801191; decimalLongitude: 141.32665; **Identification:** identifiedBy: Tatsushi Takayanagi; dateIdentified: 2020; **Event:** samplingProtocol: street light; eventDate: 2020-07-15; **Record Level:** collectionCode: Insects
- **Type status:** Other material. **Occurrence:** recordedBy: Tomiko Ito; individualCount: many; sex: male; lifeStage: adult; preparations: 99% EtOH; **Taxon:** scientificName: *Caenis rivulorum*; kingdom: Animalia; phylum: Euarthropoda; class: Insecta; order: Ephemeroptera; family: Caenidae; genus: Caenis; specificEpithet: *rivulorum*; scientificNameAuthorship: Eaton; **Location:** country: Japan; stateProvince: Hokkaido; municipality: Chitose-shi; locality: Rankoshi; decimalLatitude: 42.809656; decimalLongitude: 141.574878; **Identification:** identifiedBy: Tatsushi Takayanagi; dateIdentified: 2020; **Event:** samplingProtocol: light trap; eventDate: 2020-07-06; **Record Level:** collectionCode: Insects
- **Type status:** Other material. **Occurrence:** recordedBy: Tomiko Ito; individualCount: many; sex: female; lifeStage: adult; preparations: 99% EtOH; **Taxon:** scientificName: *Caenis rivulorum*; kingdom: Animalia; phylum: Euarthropoda; class: Insecta; order: Ephemeroptera; family: Caenidae; genus: Caenis; specificEpithet: *rivulorum*; scientificNameAuthorship: Eaton; **Location:** country: Japan; stateProvince: Hokkaido; municipality: Chitose-shi; locality: Rankoshi; decimalLatitude: 42.809656; decimalLongitude: 141.574878; **Identification:** identifiedBy: Tatsushi Takayanagi; dateIdentified: 2020; **Event:** samplingProtocol: light trap; eventDate: 2020-07-06; **Record Level:** collectionCode: Insects

#### Description

**Figure 2.**
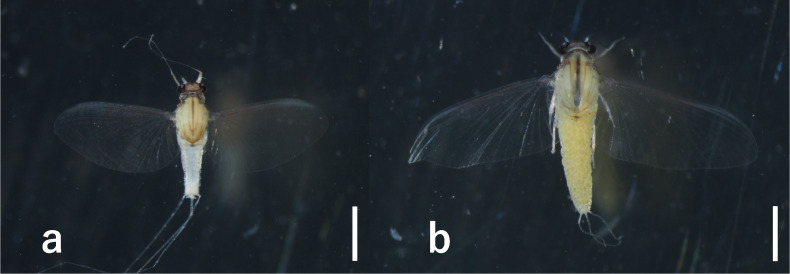
*Caenis rivulorum*: **a.** habitus, male (specimen from Loc. 1, scale: 1 mm); **b.** habitus, female (specimen from Loc. 2, scale: 1 mm).

**Figure 3.**
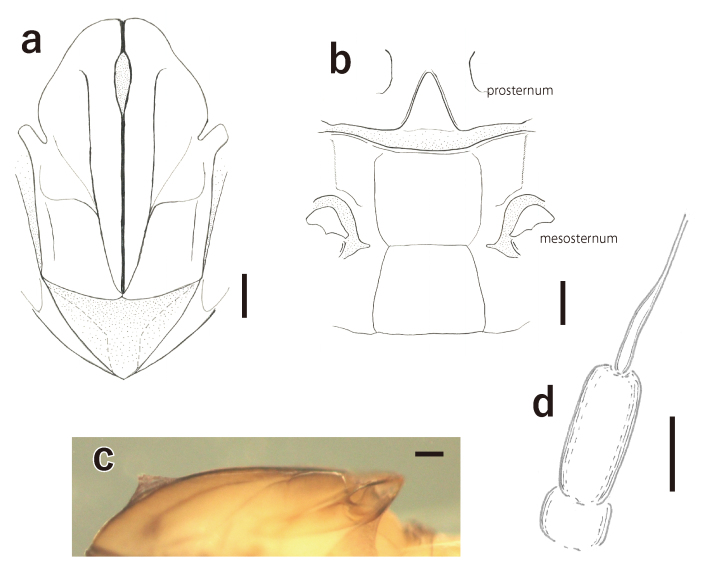
*Caenis rivulorum*: **a.** mesonotum, dorsal, male (scale: 0.1 mm); **b.** pro- and mesosternum, male (scale: 0.1 mm); **c.** mesonotum, lateral, female (scale: 0.1 mm); **d.** anntenae, female (scale: 0.05 mm).

**Figure 4.**
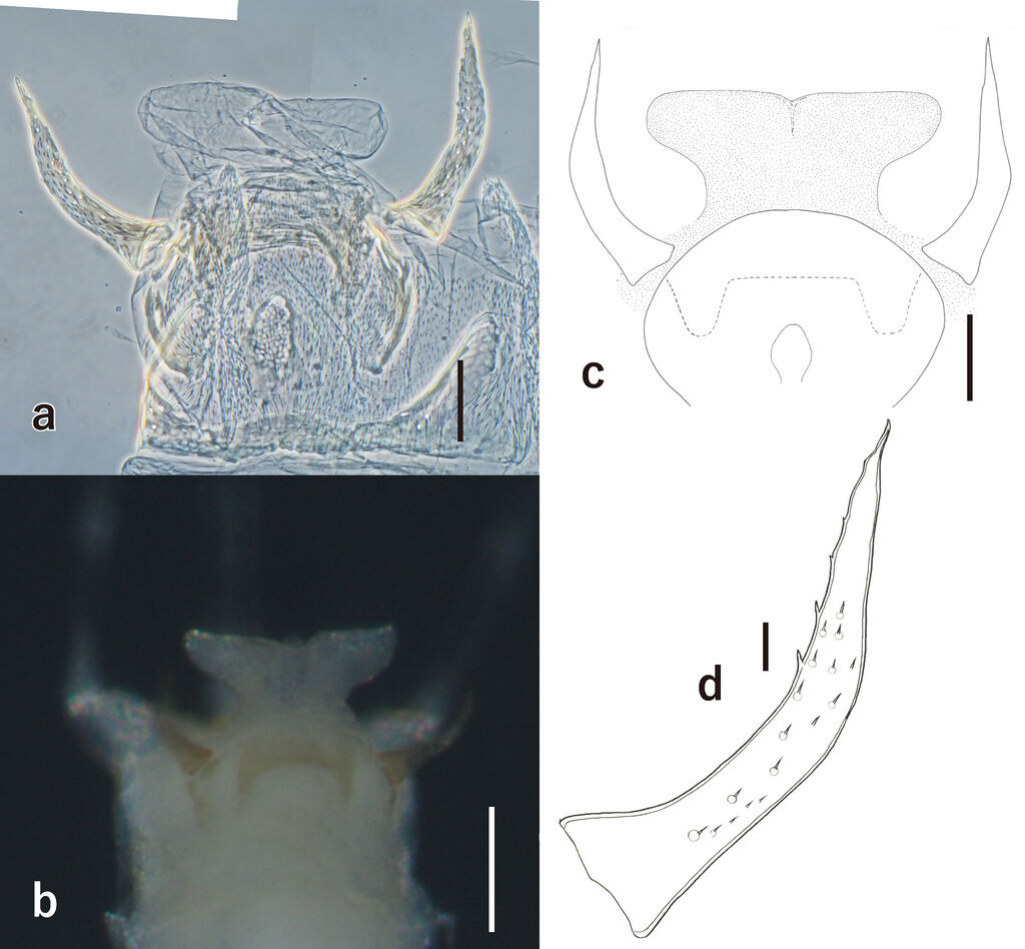
*Caenis rivulorum*: **a.** image of genital structures (ventral view, in preparation, scale: 0.05 mm); **b.** image of genital structures (ventral view, scale: 0.1 mm); **c.** line-drawing of genital structures (schematic, scale: 0.05 mm); **d.** forceps (scale: 0.01 mm).

**Male adults** (Fig. 2**a, in alcohol)**: Body length: 2.1–2.2 mm; fore-wing length: 2.3–2.4 mm; caudal filaments length: 7.0–7.8 mm. Head and prothorax dark-brown to reddish-brown. Compound eyes black. Ocelli white with black rings. Antennae white. Mesothorax brownish-white, dorsal and ventral sutures of mesothorax as in Fig. 3a and b. Fin-shaped process on mesonotum. Wings hyaline, veins of costa, subcosta and radius area slightly brownish. Legs whitish. Abdomen whitish. Forceps thin, vent in middle, with short setae on their surface (Fig. 4d). Penis anvil-shaped (Fig. 4a, b and c). Caudal filaments white.

**Female adults (Fig. 2b, in alcohol)**

Body length: 2.6–2.8 mm; fore-wing length: 2.8–3.1 mm; caudal filaments length: 0.8–1.0 mm.

Most features similar to male. Abdomen seems yellow by inner eggs and some brownish pigmentation on surfaces of each segments.

**Eggs (Fig. 5)**

Longitudinal length ca. 115 µm, latitudinal length ca. 54 µm. Surface fine-granulated, with polar caps on both ends. Micropyle widens to aperture.

#### Diagnosis

**Male adults (Fig. 2a)**

Compared to the European materials examined in Malzacher (1986), the presently examined specimens are slightly smaller.

Thorax: Prosterunum triangular (Fig. 3b). This feature is diagnostic for the genus *Caenis* to distinguish it from the other genera distributed in the Palearctic Region.

Genital characters: Malzacher (1984) classified European species of *Caenis* into two "lineages": the *horaria*-lineage and the *macrura*-lineage. The former consists of the *horaria*-group and *rivulorum*-group. The species of this lineage have straight forceps with strong tips. The species of the *macrura*-lineage have bent forceps tips with some bristles or spines. *Caenis rivulorum* has long forceps which strongly bend inwardly (Fig. 4a, b, c and d). Its penis shaft is thinner than that in *C. horaria* and the penis lobes are wider (Fig. 4a, b and c). The genital characters of the Japanese specimens agree well with those of the European specimens as described in Malzacher (1986) and Malzacher (1984).

**Female adults (Fig. 2b)**

Female adult *C. rivulorum* is similar to *C. horaria* but, by using the following characters, they can be distinguished from *C. horaria*: mesothorax with fin-shaped process (absent in *C. horaria*) (Fig. 3c), abdominal posterolateral processes short (long and filiform in *C. horaria*), base of flagellum evenly tapering (abruptly narrowing in *C. horaria*) (Bauernfeind and Soldan 2012) (Fig. 3d).

**Eggs (Fig. 5)**

The features of eggs similar to *C. horaria*. According to Malzacher (1982), eggs of *C. rivulorum* usually slimmer than those of *C. horaria* and polar caps less bulging than those of *C. horaria*. The shape of micropyle also distinguishes *C. rivulorum* from other species.

#### Molecular analysis

**Figure 6.**
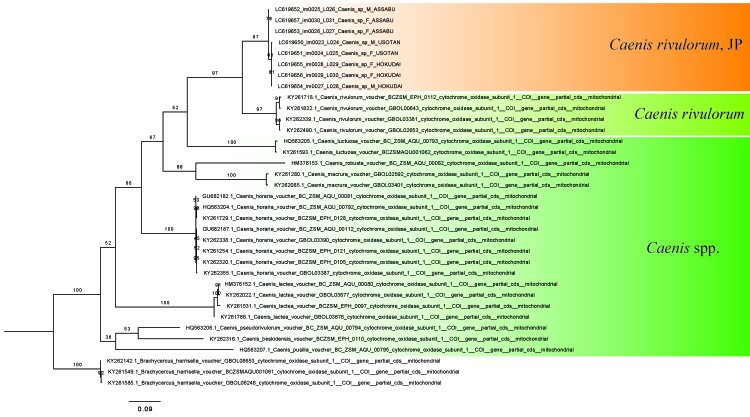
Maximum Likelihood tree of barcoding regions (partial codes of COI gene, 35 sequences, 612 bp including gaps). The numbers at branches indicate the Ultra-Fast Bootstrap values. "JP" means "Japanese" materials examined this time.

TIM2+F+I+G4 substitution model was selected as the best fit model according to BIC (Bayesian Information Criterion). By the Maximum Likelihood analysis of COI sequences, the present samples were clustered with the German *C. rivulorum* (Fig. 6). However, their pairwise genetic distance exceeded 11%. The Japanese sequenced samples showed only a little genetic variation, at most 1% by pairwise distance (Table 1).

## Discussion

### Male genital characters

The genital characters of the Japanese specimens agree well with those of the European specimens as described in Malzacher (1986) and Malzacher (1984): the caudal part of styliger roundly convex, forceps strongly bent in middle and strongly sclerotised in tip, penis anvil-shaped and widens in apex. The shape of penis lobe varies as straight rear edge to V-shaped (Malzacher 1984, Malzacher 1986), but this variation depends on the contraction of the penis-muscles (Malzacher 1991). Our specimens have straight or slightly bent rear edge as in Fig. 4a, b and c. Malzacher (1986) described the lateral-sclerite, the base of forceps and the styliger-sclerite rarely tinted brownish and our Japanse specimens show this colouration (Fig. 4b). All other articles describing the genital characters of this species also show long forceps bending inwardly and penis lobe widens in apex (Saaristo 1966, Zhou and Zheng 2004, Zhou et al. 1997, Bauernfeind and Soldan 2012) and do not contradict the Japanese specimens.

### Intraspecific genetic difference

According to the morphological observations, we identified the Japanese samples as *C. rivulorum*, as is noted in Diagnosis, Taxon treatments. However, the genetic difference between Japanese (Hokkaido) samples and European (German) *C. rivulorum* is significantly larger (> 11%) than 2.2%, which has been generally recognised as a level of divergence delimiting species across diverse insect taxa (Hebert et al. 2003). The genetic difference may indicate a possibility that they should be designated as independent species. Nevertheless, we could not find any morphological differences between the Japanese samples and the European population of *C. rivulorum*, as described in Malzacher (1982), Malzacher (1984), Malzacher (1986). Large intraspecific variations of COI gene were also identified in *Cloeon dipterum* Linnaeus, 1761, the widely distributed baetid species known to be a habitat-generalist (Rutschmann et al. 2014, Rutschmann et al. 2017, Yano et al. 2019), in which seven distinct clades showing a maximum of ca. 13% of uncorrected genetic differences were identified. Amongst them, four Eurasian clades (named "CT1", "CT2", "CT3" and "JK") show at most 9.1% ("CT3", northern Europe vs. "JK", Japan and Korea) p-distance. *Caenis rivulorum* also inhabits a wide range of habitats (Bauernfeind and Soldan 2012) and is distributed from the east to the west part of Eurasia so that, as in the case of *Cloeon dipterum*, the genetic distance can be high when compared with distantly-located populations. In the case of *Cl. dipterum*, the larger genetic distance between Eurasian clades and Macaronesian clades (named "IS1", "IS2" and "IS3") have been shown (Yano et al. 2019) and those Macaronesian populations regarded as being distinct species (Rutschmann et al. 2014, Rutschmann et al. 2017). Future detailed studies might reveal the structures of populations and speciations; however, we currently do not have any reason to separate morphologically indistinct populations as independent species, which may even cause taxonomic confusions.

### Brief review of the study of *Caenis* in Japan.

The Japanese fauna of the genus *Caenis* are poorly studied. The following two named species have been reported from Japan to date (Ishiwata 2018):

1. *Caenis horaria* (Linnaeus, 1758) from Honshu. This species was reported by Ueno and Okamoto (1932) in *Nippon Kontyu Zukan* ("Iconographia Insectrum Japonicorm"). However, only a brief description of the species was given in the article. This species is morphologically similar to *C. rivulorum*. Therefore, we need further consideration for the record of this species from Japan.
2. *Caenis nishinoae* Malzacher, 1996 from Lake Biwa. This species was described by Malzacher (1996), in which detailed descriptions of all stages of both sexes are given. This species can be clearly distinguished from *C. rivulorum*.

In addition, a couple of unidentified species have been reported, based on larval stages: *Caenis* sp. CA and *Caenis* sp. CB (Gose 1958). We can assume that those species are not *C. rivulorum* for the following reasons: the lateral margin of pronotum in *Caenis* sp. CA expands anteriorly and forms a tip apically (Gose 1958), but is straight or slightly convex and more or less parallel in *C. rivulorum* (Bauernfeind and Soldan 2012). *Caenis* sp. CB does not have short strong bristles on its fore-femora (Gose 1958) in contrast to 3–7 relatively short strong bristles on fore-femora in *C. rivulorum* (Bauernfeind and Soldan 2012).

Ishiwata (2001) reported *Caenis* sp. with a description of the adult structures, but the genital structures were not described nor illustrated. According to the drawing in Ishiwata (2001), the antennal and sternum structures are similar to those of *C. rivulorum* examined here. However, we cannot determine that *Caenis* sp. is *C. rivulorum*, based only on the published information.

Ishiwata and Fujitani (2018) described two SEM-image of eggs of unidentified species of *Caenis*: *Caenis* sp. 1 and *Caenis* sp. 2. The micropyle of those eggs is straight to aperture which is in contrast to that of *C. rivulorum*.

Recently, an additional unidentified species of the genus was reported from Ura-Bandai, Fukushima, Japan (Masubuchi and Tsutsumi 2015, Masubuchi and Tsutsumi 2014), which shows a unique shape of the male genitalia. Detailed morphological and ecological information have been provided for this species, but a taxonomic name has not yet been proposed. This species can clearly be differentiated from *C. rivulorum*, based on the male genitalia.

## Supplementary Material

XML Treatment for Caenis rivulorum

## Figures and Tables

**Figure 5.**
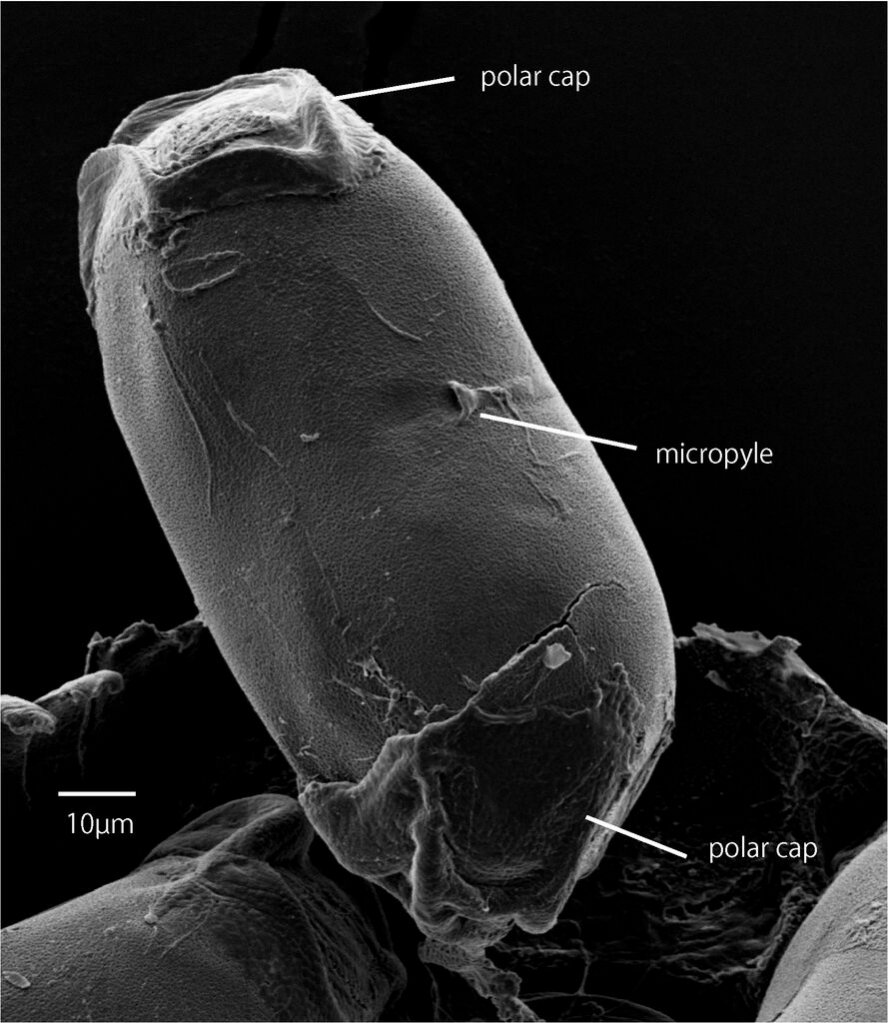
*Caenis rivulorum*, egg. SEM image, scale = 10 µm.

**Table 1.** Mean/Standard deviation/minimum-maximum values (%) of intraspecific pairwise genetic distance (p-distance) between Japanese and European samples. "JP" means "Japanese" materials examined this time.

**Species**	*C. rivulorum*, JP	*C. rivulorum*
*C. rivulorum*, JP	0.47/0.40/ 0–0.97	11.3/0.19/ 11.0–11.59
*C. rivulorum*	-	1.45/0.78/ 0.16–2.42
